# Internal Fixation *versus* Hemiarthroplasty in the Treatment of Unstable Intertrochanteric Fractures in the Elderly: A Systematic Review and Meta‐Analysis

**DOI:** 10.1111/os.12736

**Published:** 2020-07-21

**Authors:** Dong‐peng Tu, Zheng Liu, Yi‐kang Yu, Chao Xu, Xiao‐lin Shi

**Affiliations:** ^1^ Zhejiang Chinese Medical University Second Clinical Medical College of Zhejiang Chinese Medical University Zhejiang China; ^2^ Department of Orthopaedics Xinhua Hospital of Zhejiang Province, The Second Affiliated Hospital of Zhejiang Chinese Medical University Hangzhou China

**Keywords:** Elderly, Hemiarthroplasty, Hip fractures, Internal fixators, Meta‐analysis

## Abstract

**Objective:**

To evaluate the clinical efficacy of internal fixation *versus* hemiarthroplasty in the treatment of unstable intertrochanteric fractures in the elderly.

**Methods:**

A search was conducted in PubMed, Web of Science, Embase, and Cochrane Library databases up to April 2020. The present study compared internal fixation and hemiarthroplasty in the treatment of unstable intertrochanteric fractures in the elderly. RevMan5.3 software provided by the International Cochrane Group was used for the meta‐analysis. To compare the differences in the operation time, intraoperative bleeding, length of hospital stay, superficial infection, Harris hip score, mortality within 1 year, mortality within 2 years, reoperation, incidence of deep venous thrombosis (DVT), partial weight‐bearing time, non‐union, and implant‐related complications between an internal fixation group and an hemiarthroplasty group.

**Results:**

A total of 1300 patients were included in nine studies. The results showed that the operation time (*MD* = −18.09, 95% *CI*: −27.85–−8.34, *P* = 0.0003), intraoperative bleeding (*MD* = −195.31, 95% *CI*: −244.8–−147.74, *P* < 0.0001), implant‐related complications (*MD* = 3.83, 95% *CI*: 1.74–8.45, *P* = 0.0008), and partial weight‐bearing time (*MD* = 17.21, 95% *CI*: 1.63–32.79, *P* = 0.03) have statistical significance. However, there is not statistical significance for the Harris hip joint function scale (HHS) (*MD* = 5.60, 95% *CI*: −1.13–12.33, *P* = 0.10), DVT (*MD* = 1.02, 95% *CI*: 0.45–2.27, *P* = 0.97), length of hospital stay (*MD* = ‐1.08, 95% *CI*: −2.82–0.66, *P* = 0.22), superficial Infection (*OR* = 0.92, 95% *CI*: 0.43–1.98, *P* = 0.89), mortality within 1 year (*OR* = 0.95, 95% *CI*: 0.61–1.48, *P* = 0.81), mortality within 2 years (*OR* = 0.93, 95% *CI*: 0.61–1.43, *P* = 0.75), reoperation (*MD* = 1.80, 95% *CI*: 0.64–5.04, *P* = 0.26), and non‐union (*OR* = 1.20, 95% *CI*: 0.48–3.03, *P* = 0.70). The result of the subgroup analysis showed no significant differences between the less than 2 years follow‐up and the 2 years or more follow‐up group. The only difference was for the Harris hip score: the internal fixation group was superior to the hemiarthroplasty group in the less than 2 years subgroup analysis, while there was no difference between the internal fixation group hemiarthroplasty group in the 2 years or more subgroup analysis.

**Conclusion:**

Compared with the internal fixation group, those in the hemiarthroplasty group could carry out weight‐bearing training early and implant‐related complications were reduced, but it requires longer operation time and there is greater intraoperative blood loss. There is no difference in mortality, the incidence of DVT, non‐union, HHS, reoperation, length of hospital stay, and superficial infection. Hemiarthroplasty may be a better choice for unstable intertrochanteric fractures in the elderly.

## Introduction

Hip fractures are common and are a huge economic burden on the healthcare system. The morbidity of intertrochanteric fractures is rising and the number of hip fractures worldwide could reach 4.5 mn by 2050^1^. Aging and osteoporosis are the main causes of hip fractures. In an epidemiological investigation in Spain of 4415 patients with hip fractures, 4271 (3346 females, 925 males) were diagnosed with osteoporosis^2^. With osteoporosis, the trabeculae become thinner and sparse, the bone cortex becomes thinner, and the bone strength decreases. Slight trauma can easily result in fracture. Once a femoral intertrochanteric fracture occurs, a comminuted fracture can easily occur^3^.

Femoral intertrochanteric fractures account for approximately half of all hip fractures. Intertrochanteric fractures can be divided into stable and unstable fractures based on AO/OTA or Evans–Jensen classification. A2, A3, or Evans–Jensen III, IV, and V are considered unstable intertrochanteric fractures. For unstable intertrochanteric fractures in the elderly, conservative treatment can lead to complications, such as bedsores, deep venous thrombosis (DVT), pendant pneumonia, and death. According to clinical trials, surgical treatment has the advantages of stable fixation and getting out of bed sooner, so it has gradually become the first choice in the clinic^4^. Although the best surgical treatment has always been controversial, the main aims are early rehabilitation and return to social activities. Internal fixation and hip replacement are the main surgical treatments. Internal fixation includes use of cephalomedullary nails, proximal femoral nail antirotation, proximal femoral nails, gamma nails, dynamic hip screws, and compression hip screws. Hip replacement includes application of hemiarthroplasty and total hip arthroplasty. Camurcu^5^ points out that hemiarthroplasty has the advantages of early mobilization, acceptable functional results, and lower failure rates in the treatment of unstable intertrochanteric fractures in elderly patients. Kumar *et al*. conducted a meta‐analysis of proximal femoral nails *versus* hemiarthroplasty in the treatment of unstable intertrochanteric fractures^6^ and Li *et al*. conducted a meta‐analysis of internal fixation *versus* external fixation in the treatment of unstable interochanteric^7^. However, there is no meta‐analysis comparing internal fixation *versus* hemiarthroplasty in the treatment of unstable intertrochanteric fractures in the elderly. We conducted a meta‐analysis to compare the clinical efficacy and safety of internal fixation and hemiarthroplasty in the treatment of unstable intertrochanteric fractures in the elderly. The outcome indicators include operation time, intraoperative bleeding, length of hospital stay, superficial infection, Harris hip joint function scale (HHS), mortality within 1 year, mortality within 2 years, reoperation, DVT, partial weight‐bearing time, implant‐related complications, and non‐union.

## Materials and Methods

The Preferred Reporting Items for Systematic Reviews and Meta‐Analyses (PRISMA) statement was used to guide the study^8^.

### *Information Sources and Search Strategy*

We searched four electronic databases up to April 2020: Embase, Pubmed, Web of Science, and Cochrane Library. We used mesh and free terms to search the electronic databases, Medical Subject Headings (MeSH terms) “hemiarthroplasty” and free terms, MeSH terms “hip fractures” and free terms, MeSH terms “internal fixators” and free terms: (Hemiarthroplasty OR hemiarthroplasties OR hemiarthroplasty OR hemi‐arthroplasties) AND (hip fractures OR intertrochanteric fractures OR intertrochanteric femoral fracture OR trochanteric fracture) AND (internal fixators OR internal fixation OR internal fixator OR cephalomedullary nail OR proximal femoral nail antirotation OR proximal femoral nail OR intramedullary nail OR gamma nail OR InterTAN nail OR DHS OR dynamic hip screw OR CHS OR compression hip screw OR plate).

### *Inclusion Criteria and Exclusion Criteria*

The inclusive selection criteria are as follows:

1. Elderly patient (patient ≥60 years old) with unstable intertrochanteric fracture.
2. Participants were treated with internal fixation, such as cephalomedullary nail, proximal femoral nail antirotation, proximal femoral nail, gamma nail, INTERTAN nail, dynamic hip screw, compression hip screw, or plate.
3. Participants were treated with hemiarthroplasty.
4. Operation time (minute), intraoperative bleeding (mL), length of hospital stay (days), superficial infection, Harris hip score, mortality within 1 year, mortality within 2 years, reoperation, DVT, partial weight‐bearing time (days), non‐union, and implant‐related complications.
5. Retrospective comparative control trial, randomized controlled trials, or cohort study.

The exclusion criteria were as follows: (i) age <60 years old; (ii) stable intertrochanteric fracture; (iiii) pathological fracture; (iv) open fracture; (v) repeatedly published article; (vi) non‐English literature; (vii) average follow‐up time less than 12 months; and (viii) low quality literature.

### *Data Extraction*

Data includes general information and clinical outcomes. General information includes author, year of publication, study design, average age, gender, type of fracture, and average follow‐up time. Clinical outcomes include operation time (min), intraoperative bleeding (mL), length of hospital stay (days), superficial infection, HHS, mortality within 1 year, mortality within 2 years, reoperation, DVT, partial weight‐bearing time (days), implant‐related complications, and non‐union.

### *Risk of Bias Assessment*

All the literature was screened by two analysts according to the inclusion and exclusion criteria. When the two analysts had different opinions, they would ask the third analyst for their opinion. If the study was a random controlled trial (RCT), the Cochrane Collaboration tool was used for evaluation. The Cochrane Collaboration tool has seven domains for assessment: random sequence generation, allocation concealment, blinding of participants and personnel, blinding of outcome assessment, incomplete, outcome, selective outcome reporting, and other sources of bias. The risk of bias includes three types: low risk, high risk, or unclear risk. The quality of the non‐RCT was assessed with the Newcastle–Ottawa scale (NOS). It contains eight items, which are categorized into three dimensions: selection, comparability, and exposure (case‐control study) or outcome (cohort study); a maximum of 4 stars could be given in “selection,” a maximum of 2 stars could be given in “comparability,” a maximum of 4 stars could be given in “exposure or outcome.” One star equated to 1 point, the full score was 10 points, and it was classified as low‐quality literature when NOS score ≤ 5 points. The higher the score, the better the quality of the literature.

### *Statistical Analysis*

RevMan 5.3 software was used for data analysis. The odds ratios (*OR*) represent the continuous variable; weighted mean differences (WMD) represent a dichotomous variable. Both were assessed with 95% confidence intervals. The *I* ^2^‐value and the χ^2^‐test were used to assess the heterogeneity; if the heterogeneity was small (*P* > 0.1, *I* ^2^ ≤ 50%), a fixed effect model was used. If the heterogeneity was large (*P* < 0.1, *I* ^2^ > 50%), a random effect model was used. The forest plot was used to show the results of the meta‐analysis. It was considered statistically significant when the *P*‐value was less than 0.05.

## Result

### *Literature Screening*

**Fig 1:**
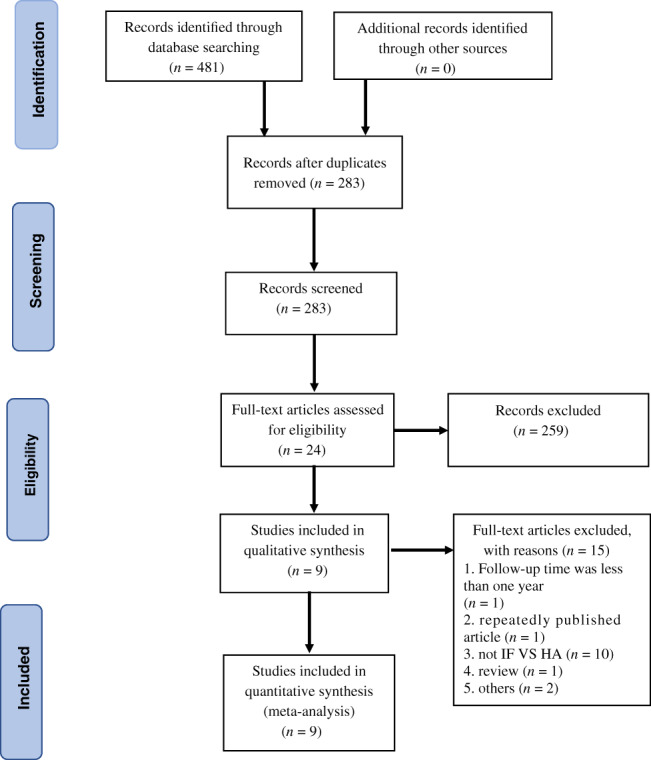
Preferred reporting items for systematic reviews and meta‐analyses flow diagram of study selection.

The literature was screened strictly by two evaluators by reading the title, abstract, or full text of the article according to the inclusion and exclusion criteria. In the process of extracting the data, the two researchers would recheck it again and again; if there were any differences, the third evaluator would assist. A total of 481 English language studies was preliminarily obtained from the database; 198 were duplicate articles and 259 articles were removed after reading the title or abstract. The full text of 24 articles was read and, finally, nine articles^9^, ^10^, ^11^, ^12^, ^13^, ^14^, ^15^, ^16^, ^17^ were included in this study (Fig. 1). There were a total of 1300 patients, forming the IF group (*n* = 776) and the hemiarthroplasty group (*n* = 524). General information on the nine studies is shown in Table 1.

**TABLE 1:** General information of included studies

Study	Year	Study design	Compassion	Number	Age (years)	Female/male	Outcome	Fracture classification	Follow‐up (months)
Elhadi^9^	2018	PCS	IF(PFN,DHS)	57	77.2 ± (65–105)	31:26	HJKLM	AO/OTA 31.A2.2.A2.3	13.6
			HA	60	76.1(65–91)	37:23			12
Jolly^10^	2019	RCT	IF(PFN)	50	75(75–85)	unclear	ABDFHJKL	Unstable (unclear)	12
			HA	50	75(75–85)	unclear			12
Kayali^11^	2006	CCT	IF(Unclear)	45	75 ± 6	24:21	ACJK	AO/OTA 31.A2.1‐A3.3	29
			HA	42	73 ± 9	31:12			24
Kim^12^	2020	CCT	IF(NAIL,DHS)	396	79.5 ± (65–102)	276:120	FGH	AO/OTA 31.A2.1.A3.3	29
			HA	168	83 ± (65–102)	136:32			28
Kim^13^	2005	RCT	IF(PFN)	29	81 ± 3.2	21:8	ABCDFHJKLM	Unstable (unclear)	36
			HA	29	82 ± 3.4	21:6			36
Kim^14^	2014	CCT	IF(CHS)	43	75.5 ± 6.5	31:11	ACEJKM	AO/OTA 31.A2.	25
			HA	46	79.7 ± 6.5	41:5			30
Park^15^	2015	CCT	IF(Gamma，PFN，PFNA)	31	78.1(73–86)	19:12	ABCDFGHJM	AO/OTA 31.A3	24
			HA	22	76.9(73–84)	18:4			24
Shen^16^	2012	CCT	IF(NAIL,DHS)	64	76.8(70–98)	48:16	ABFGHJKLM	AO/OTA 31.A2.2‐A3.3	24
			HA	60	78.2(70–101)	47:13			24
Zhou^17^	2019	CCT	IF(PFNA)	61	83.5 ± 4.8	25:36	ABCDEL	Evens–Jensen III IV V	28
			HA	47	83.8 ± 6.4	20:27			28

A, operation time; B, intraoperative bleeding volume; C, length of hospital stay; CCT, retrospective comparative control trial; CHS, compression hip screw; D, Harris hip joint function scale; DHS, dynamic hip screw; E, partial weight‐bearing time; F, mortality within 1 year; G, mortality within 2 years; H, reoperation; HA, hemiarthroplasty; IF, internal fixation; J, implant‐related complications; K, superficial infection; L, deep venous thrombosis; M, non‐union; NAIL, intramedullary nail (unclear); PCS, prospective cohort study; PFN, proximal femoral nails; PFNA, Proximal Femoral Nail Antirotation; RCT, randomized controlled trials.

### *Quality Assessment of the Eligible Studies*

**Fig 2:**
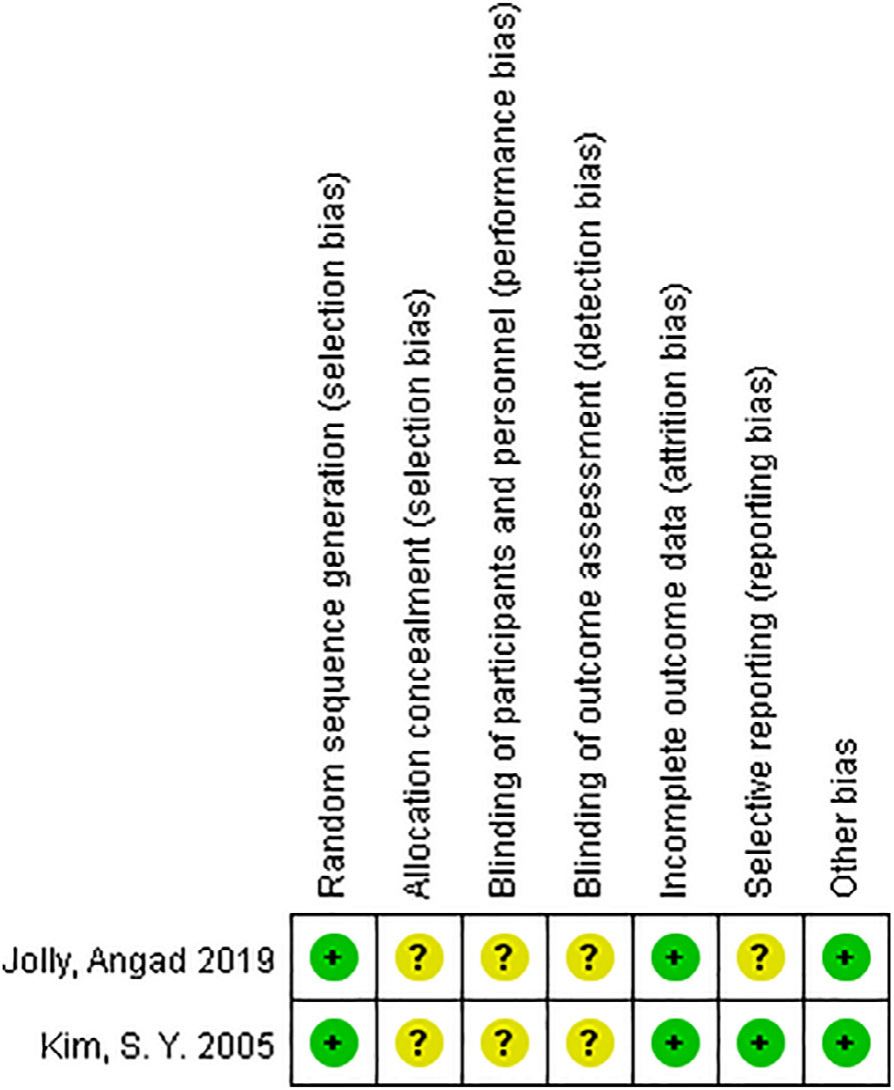
Risk of bias assessment summary of randomized controlled trials.

Two reviewers independently used the Cochrane Collaboration tool for assessing risk bias or used the Newcastle–Ottawa scale (NOS) to assess the quality of the eligible studies: two randomized controlled trials^10^, ^13^, one prospective cohort study^9^, and six retrospective studies^11^, ^12^, ^14^, ^15^, ^16^, ^17^. Risk of bias assessment of RCT was assessed using the Cochrane Collaboration tool. The quality of the RCT were accepted: one has three low risks bias, the other has four low risks bias，Jolly^10^ and kim^13^ did not describe allocation concealment, blinding of participants and personnel, blinding of outcome assessment, kim^13^ did not describe selective outcome reporting (Fig. 2). The quality of the non RCT was assessed with the Newcastle–Ottawa scale. Four studies scored 6 points, two studies scored 7 points, and one study scored 8 points (Table 2).

**TABLE 2:** Quality assessment of non‐randomized controlled trials (Newcastle–Ottawa scale for non‐randomized controlled trials)

Study	Selection	Comparability	Exposure or Outcome	total score
Elhadi (2018)^9^	**★★**	**★★**	**★★★**	**7**
Kayali (2006)^11^	**★★★**	**★**	**★★**	**6**
Kim (2020)^12^	**★★**	**★★**	**★★**	**6**
Kim (2014)^14^	**★★★**	**★**	**★★**	**6**
Park (2015)^15^	**★★**	**★★**	**★★**	**6**
Shen (2012)^16^	**★★**	**★★**	**★★★**	**7**
Zhou (2019)^17^	**★★★**	**★★**	**★★★**	**8**

### *Intraoperative Bleeding Volume (mL)*

**Fig 3:**
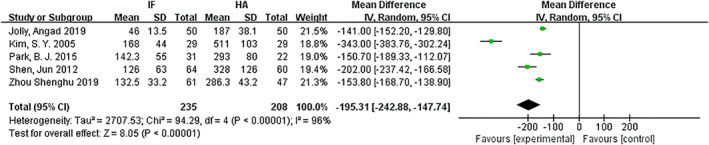
Forest plot diagram of intraoperative bleeding volume compared between internal fixation and hemiarthroplasty.

Intraoperative blood loss was compared for internal fixation and hemiarthroplasty. Only five articles^10^, ^13^, ^15^, ^16^, ^17^ mentioned the amount of intraoperative blood loss and included mean and standard deviation. The results of the fixed effect model show that there is statistical heterogeneity among studies (*P* < 0.00001, *I* ^2^ = 96%); the random effect model was then used for analysis. The average intraoperative blood loss of the internal fixation group and the hemiarthroplasty group was 118 mL *versus* 306 mL. The results showed that the amount of intraoperative bleeding in the internal fixation group was significantly less than that in the hemiarthroplasty group (*MD* = −195.31, 95% *CI*: −244.8–−147.74, *P* < 0.0001) (Fig. 3).

### *Operation Time (Min)*

**Fig 4:**
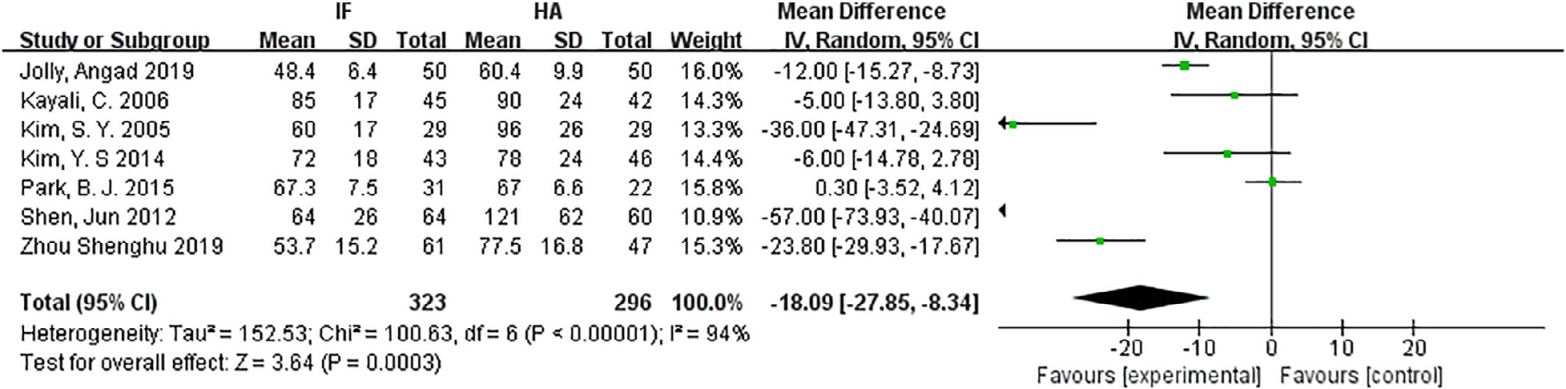
Forest plot diagram of operation time compared between internal fixation and hemiarthroplasty.

The operation times for internal fixation and hemiarthroplasty were compared. Seven articles^10^, ^11^, ^13^, ^14^, ^15^, ^16^, ^17^mentioned operation time and included mean and standard deviation. The results of the fixed effect model show that there is statistical heterogeneity among studies (*P* < 0.00001, *I* ^2^ = 94%) the random effect model was then used for analysis. The average operation time of the internal fixation group and the hemiarthroplasty group was 63.5 min *versus* 86.3 min. The results showed that the operation time in the internal fixation group was significantly less than that in the hemiarthroplasty group (*MD* = −18.09, 95% *CI*: −27.85–−8.34, *P* = 0.0003) (Fig. 4).

### *Length of Hospital Stay (Days)*

**Fig 5:**
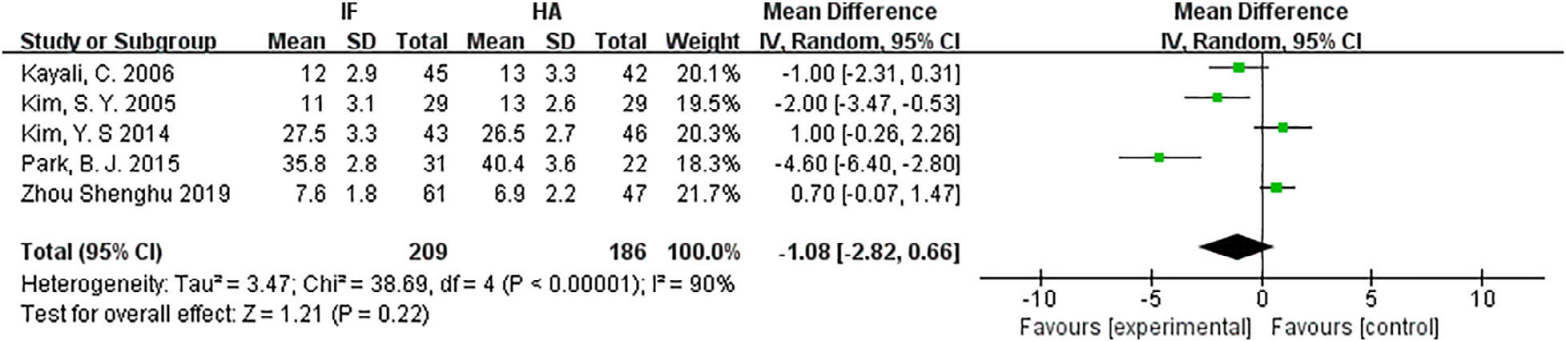
Forest plot diagram of the length of hospital stay compared between internal fixation and hemiarthroplasty.

Length of hospital stay was compared for internal fixation and hemiarthroplasty. Only five articles^11^, ^13^, ^14^, ^15^, ^17^ mentioned the hospital stay and included mean and standard deviation. The results of the fixed effect model showed that there is statistical heterogeneity among studies (*P* < 0.00001, *I* ^2^ = 90%); the random effect model was then used for analysis. The results showed that there was no significant difference in length of hospital stay between the internal fixation group and hemiarthroplasty groups (*MD* = −1.08, 95% *CI*: −2.82–0.66, *P* = 0.22) (Fig. 5).

### *Harris Hip Joint Function Scale*

**Fig 6:**
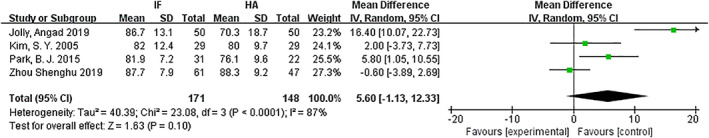
Forest plot diagram of compared Harris hip joint function scale between internal fixation and hemiarthroplasty.

The HHS at the end of follow up was compared for the internal fixation and the hemiarthroplasty. Only four articles^10^, ^13^, ^15^, ^17^ mentioned HHS and included mean and standard deviation. The results of the fixed effect model show that there is statistical heterogeneity among studies (*P* < 0.00001, *I* ^2^ = 87%); the random effect model was then used for analysis. The results showed that there was no significant difference in Harris hip score at the end of follow up between the internal fixation group and the hemiarthroplasty group (*MD* = 5.60, 95% *CI*: −1.13–12.33, *P* = 0.10) (Fig. 6).

### *Partial Weight‐Bearing Time (Days)*

**Fig 7:**
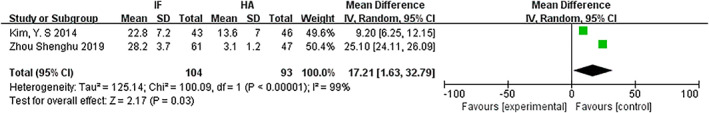
Forest plot diagram of compared partial weight‐bearing between internal fixation and hemiarthroplasty.

The partial weight‐bearing times for internal fixation and hemiarthroplasty were compared. Only two articles^14^, ^17^ mentioned the partial weight‐bearing time and included mean and standard deviation. The results of the fixed effect model show that there is statistical heterogeneity among studies (*P* < 0.00001, *I* ^2^ = 99%); the random effect model was then used for analysis. The average partial weight‐bearing time of the internal fixation group and the hemiarthroplasty group was 25.9 days *versus* 8.29 days. The results showed that the time of partial weight‐bearing in the internal fixation group was later than that in the hemiarthroplasty group (*MD* = 17.21, 95% *CI*: 1.63–32.79, *P* = 0.03) (Fig. 7).

### *Mortality within 1 year*

**Fig 8:**
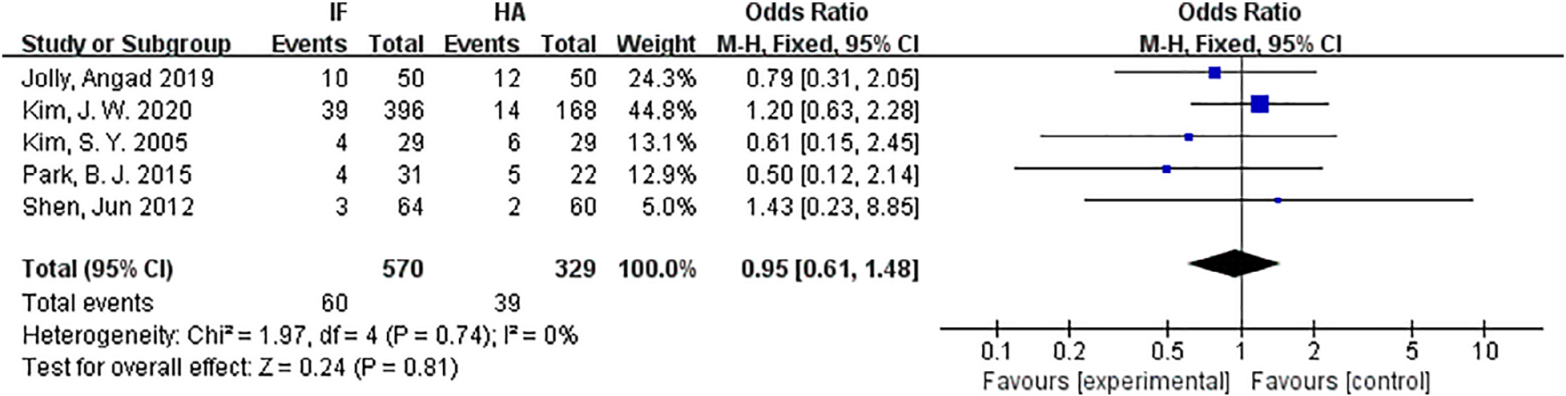
Forest plot diagram of compared mortality within 1 year between internal fixation and hemiarthroplasty.

Mortality within 1 year was compared for the internal fixation and hemiarthroplasty. There were seven articles^10^, ^12^, ^13^, ^15^, ^16^ with references to mortality. The results of the fixed effect model show that there was no statistical heterogeneity among studies (*P* = 0.74, *I* ^2^ = 0%). The results showed that there was no significant difference in mortality between the internal fixation group and the hemiarthroplasty group (*OR* = 0.95, 95% *CI*: 0.61–1.48, *P* = 0.81) (Fig. 8).

### *Mortality within 2 years*

**Fig 9:**
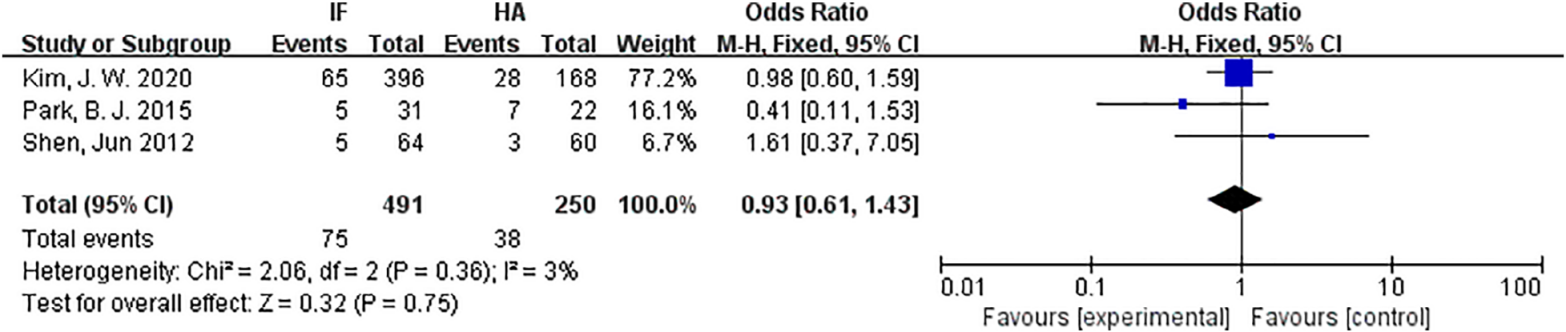
Forest plot diagram of compared mortality within 2 years between internal fixation and hemiarthroplasty.

Mortality within 2 years was compared for internal fixation and hemiarthroplasty. There were three articles^12^, ^15^, ^16^ with references to mortality. The results of the fixed effect model show that there was no statistical heterogeneity among studies (*P* = 0.36, *I* ^2^ = 3%). The results showed that there was no significant difference in mortality within 2 years between the internal fixation group and the hemiarthroplasty group (*OR* = 0.93, 95%* CI*: 0.61–1.43, *P* = 0.75) (Fig. 9).

### *Reoperation*

**Fig 10:**
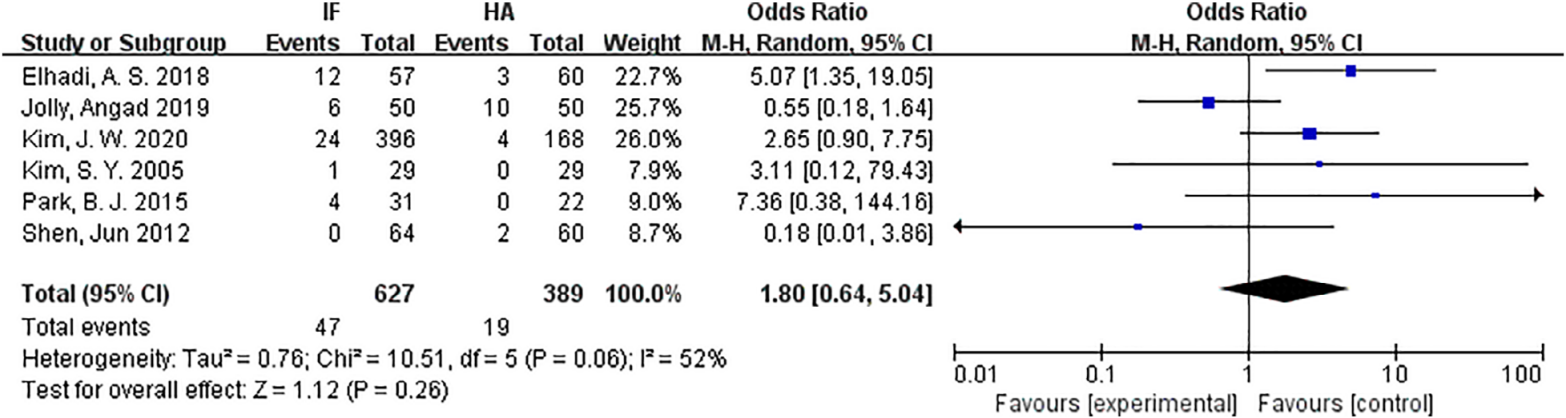
Forest plot diagram of compared reoperation between internal fixation and hemiarthroplasty.

Reoperation was compared for internal fixation and hemiarthroplasty. Six articles^9^, ^10^, ^12^, ^13^, ^15^, ^16^ mentioned reoperation. The results of the fixed effect model show that there is statistical heterogeneity among studies (*P* = 0.06, *I* ^2^ = 52%); the random effect model was then used for analysis. The results showed that there was no significant difference in reoperation between the internal fixation group and the hemiarthroplasty group (*OR* = 1.80, 95% *CI*: 0.64–5.04, *P* = 0.26) (Fig. 10).

### *Implant‐Related Complications*

**Fig 11:**
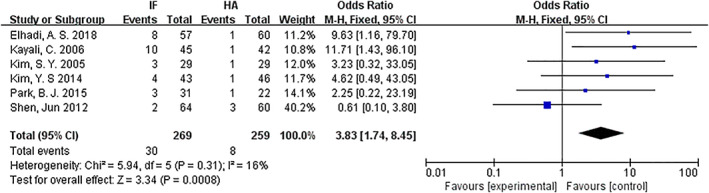
Forest plot diagram of compared implant‐related complications between internal fixation and hemiarthroplasty.

Implant‐related complications were compared for internal fixation and hemiarthroplasty. Six articles^9^, ^11^, ^13^, ^14^, ^15^, ^16^ mentioned implant‐related complications. The results of the fixed effect model show that there was no statistical heterogeneity among studies (*P* = 0.31, *I* ^2^ = 16%). The implant‐related complications of the internal fixation group and the hemiarthroplasty group were 11.1% *versus* 3%. The results showed that the implant‐related complications in the internal fixation group were significantly higher than those in the hemiarthroplasty group (*OR* = 3.83, 95% *CI*: 1.74–8.45, *P* = 0.0008) (Fig. 11).

### *Deep Venous Thrombosis*

**Fig 12:**
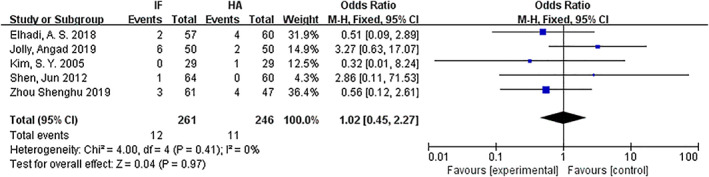
Forest plot diagram of compared deep venous thrombosis between internal fixation and hemiarthroplasty.

The DVT for internal fixation and hemiarthroplasty were compared. Five articles^9^, ^10^, ^13^, ^16^, ^17^ mentioned DVT. The results of the fixed effect model show that there was no statistical heterogeneity among studies (*P* = 0.41, *I* ^2^ = 0%). The results showed that there was no significant difference in DVT between the internal fixation group and the hemiarthroplasty group (*OR* = 1.02, 95% *CI*: 0.45–2.27, *P* = 0.97) (Fig. 12).

### *Superficial Infection*

**Fig 13:**
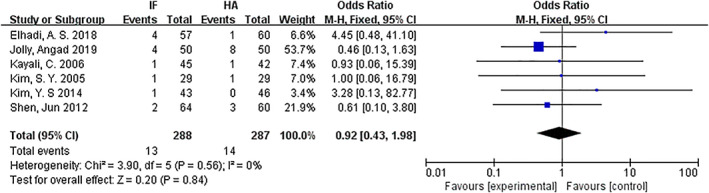
Forest plot diagram of compared superficial infection between internal fixation and hemiarthroplasty.

Superficial infection was compared for internal fixation and hemiarthroplasty. Six articles^9^, ^10^, ^11^, ^13^, ^14^, ^16^ mentioned superficial wound infection. The results of the fixed effect model show that there was no statistical heterogeneity among studies (*P* = 0.56, *I* ^2^ = 0%). The results showed that there was no significant difference in superficial infection between the internal fixation group and the hemiarthroplasty group (*OR* = 0.92, 95% *CI*: 0.43–1.98, *P* = 0.89) (Fig. 13).

### *Non‐union*

**Fig 14:**
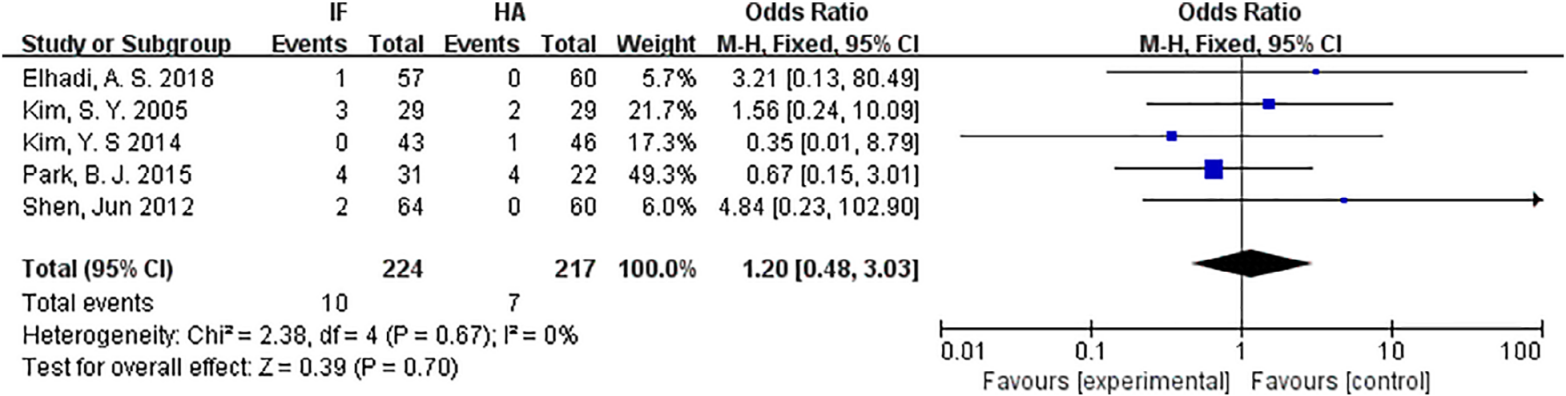
Forest plot diagram of compared non‐union between internal fixation and hemiarthroplasty.

The non‐union rate was compared for internal fixation and hemiarthroplasty. Five articles^9^, ^13^, ^14^, ^15^, ^16^ mentioned non‐union. The results of the fixed effect model show that there was no statistical heterogeneity among studies (*P* = 0.67, *I* ^2^ = 0%). The results showed that there was no significant difference in non‐union between the internal fixation group and the hemiarthroplasty group (*OR* = 1.20, 95% *CI*: 0.48–3.03, *P* = 0.70) (Fig. 14).

### *Subgroup Analysis*

We performed subgroup analysis based on average follow‐up time. The included studies were classified into less than 2 years and 2 years or more subgroups. The combined results of operation time, intraoperative bleeding, superficial infection, Harris hip score, mortality within 1 year, reoperation, DVT, non‐union, and implant‐related complications are shown in Table 3. Because the study with less than 2 years follow up did not have the outcomes for mortality within 2 years, partial weight‐bearing time, and length of hospital stay, the outcomes were not included in the subgroup analysis. In the less than 2 years subgroup analysis, the combined results showed that the internal fixation group was superior to the hemiarthroplasty group in operation time, intraoperative bleeding, and Harris hip score; the hemiarthroplasty group was superior to the internal fixation group in implant‐related complications. There is no difference in superficial infection, operation time, Harris hip score, mortality within 1 year, reoperation, DVT, non‐union, and implant‐related complications. In the 2 years or more subgroup analysis, the combined results showed that the internal fixation group was superior to the hemiarthroplasty group in operation time and intraoperative bleeding; the hemiarthroplasty group was superior to the internal fixation group in implant‐related complications. There is no difference in superficial infection, Harris hip score, mortality within 1 year, DVT, and non‐union.

**TABLE 3:** The combined results of subgroup analysis based on average follow‐up time

Outcome	Average follow‐up time	Included studies	Number	*I* ^2^	Statistic effect model	Effect estimate	*P*‐value
Intraoperative bleeding	Less than 2 years	1	100	0%	*MD* (fixed, 95% *CI*)	−4.90 [−5.69, −4.10]	<0.00001
	2 years or more	4	343	92%	*MD* (random, 95% *CI*)	−3.11 [−4.25, −1.97]	<0.00001
Operation time	Less than 2 years	1	100	0%	*MD* (fixed, 95% *CI*)	−12.00 [−15.27, −8.73]	<0.00001
	2 years or more	6	519	95%	*MD* (random, 95% *CI*)	−20.01 [−33.92, −6.09]	<0.00001
HHS	Less than 2 years	1	100	0%	*MD* (fixed, 95% *CI*)	16.40 [10.07, 22.73]	<0.00001
	2 years or more	3	219	58%	*MD* (random, 95% *CI*)	2.13 [−1.87, 6.14]	0.30
Mortality within 1 year	Less than 2 years	1	100	0%	*OR* (fixed, 95% *CI*)	0.79 [0.31, 2.05]	0.63
	2 years or more	4	799	0%	*OR* (fixed, 95% *CI*)	1.00 [0.60, 1.65]	0.99
Reoperation	Less than 2 years	2	217	85%	*OR* (random, 95% *CI*)	1.61 [0.18, 14.41]	0.67
	2 years or more	4	799	10%	*OR* (fixed, 95% *CI*)	2.22 [0.97, 5.09]	0.06
Implant‐related complications	Less than 2 years	1	117	0%	*OR* (fixed, 95% *CI*)	9.63 [1.16, 79.70]	0.04
	2 years or more	5	411	16%	*OR* (fixed, 95% *CI*)	3.10 [1.31, 7.36]	0.01
Deep venous thrombosis	Less than 2 years	2	217	56%	*OR* (random, 95% *CI*)	1.29 [0.23, 7.10]	0.77
	2 years or more	3	290	0%	*OR* (fixed, 95% *CI*)	0.69 [0.20, 2.31]	0.54
Superficial infection	Less than 2 years	2	217	68%	*OR* (random, 95% *CI*)	1.18 [0.13, 10.84]	0.88
	2 years or more	4	358	0%	*OR* (fixed, 95% *CI*)	0.97 [0.29, 3.21]	0.96
Non‐union	Less than 2 years	1	117	0%	*OR* (fixed, 95% *CI*)	3.21 [0.13, 80.49]	0.48
	2 years or more	4	324	0%	*OR* (fixed, 95% *CI*)	1.08 [0.41, 2.86]	0.88

CI, confidence interval; HHS, Harris hip joint function scale; MD, mean difference; OR, odds ratio

## Discussion

Unstable intertrochanteric fractures account for approximately 50% of femoral intertrochanteric fractures. Conservative treatment of unstable intertrochanteric fractures in the elderly will result in a variety of complications and high mortality rates. Surgical treatment can reduce the incidence of complications and death^18^. Controversy exists about the best surgical treatment for unstable intertrochanteric fractures in the elderly^4^. Some authors believe that internal fixation is associated with higher complications in the treatment of unstable intertrochanteric fractures in the elderly. Joint replacement is a better option for unstable intertrochanteric fractures in the elderly^19^. Internal fixation and hemiarthroplasty are widely used in unstable intertrochanteric fractures in the elderly. Internal fixation includes all kinds of plates, dynamic hip screws, and various intramedullary nails. In this meta‐analysis, we evaluated the clinical outcome of elderly patients with unstable intertrochanteric fractures who underwent internal fixation or hemiarthroplasty.

In this meta‐analysis, the operation time and intraoperative blood loss was significantly less in the internal fixation group than in the hemiarthroplasty group. However, in terms of implant‐related complications and partial weight‐bearing time, the hemiarthroplasty group was significantly better than the internal fixation group. There was no difference in Harris hip score, hospital stay, mortality within 1 year, mortality within 2 years, reoperation, superficial infection, non‐union rate, and DVT incidence between the two groups.

Operation time and intraoperative bleeding are important indexes of surgery. The operation time is proportional to the intraoperative blood loss. Long operation time means longer anesthesia time, which will increase the probability of gastrointestinal and respiratory system‐related complications. Operation time and intraoperative blood loss were significantly less for internal fixation than for hemiarthroplasty, but the heterogeneity was relatively large. The selection of internal fixation and the technique of the clinician may be the main reasons for the high heterogeneity. Yang^20^ points out that the percutaneous compression plate has more intraoperative blood loss and longer operation time than Gamma3. Prolongation of operation time and increase in intraoperative bleeding will increase the risk of wound infection. Wound infection is related to gender, body mass index, and surgeon's level of experience^21^. Wound infection includes superficial and deep infection. In this article, both groups have 6 cases of deep infection; there were 13 cases of superficial infection in the internal fixation group and 14 cases in the hemiarthroplasty group. There was no difference in the risk of superficial and deep infection between the internal fixation group and the hemiarthroplasty group. In Jolly *et al*., the infection rate in the hemiarthroplasty group (24%) was significantly higher than that in the internal fixation group (8%)^10^. If the wound is infected, this will lead to a longer stay in hospital and increase the cost of hospitalization. Kim^11^ points out that the clinical cost of the hemiarthroplasty group was significantly higher than that of the internal fixation group. In Wang's^22^ survey of the clinical cost of hip fractures in China, the cost of joint replacement is slightly higher than that of internal fixation. The cost of hip fracture has become a considerable burden for patients. There is no significant difference in hospital stay between the internal fixation group and hemiarthroplasty group, but the heterogeneity is great. Despite the people in the study all being over 60 years old, the age range is relatively large. Some have more physical diseases and stay in the hospital for a longer time. Yoo^23^ points out that the hospital stay was short (less than 10 days), and the mortality rate was as high as 21.7% 1 year later; when hospital stay was 11–20 days, the mortality was only 12.4% 1 year later. In this meta‐analysis, there was no significant difference in mortality within 1 year and within 2 years between the internal fixation group and the hemiarthroplasty group. Morri^24^ points out that the main causes of death within 1 year of hip fracture were older age, more comorbidities, and physical weakness.

The degree of hip function recovery affects patients' quality of life. The commonly used score for hip function is the HHS. The higher the score, the better the recovery of hip function^25^. The HHS is a rating scale with a total of 100 points (excellent, ≥90 points; good, 80–89 points; fine, 70–79 points; and poor, <70 points): it includes deformity, motion, the domains of pain, and function. In the comparison of the HHS, there was no significant difference between the two groups. In the less than 2 years subgroup analysis, the internal fixation group was superior to hemiarthroplasty group in the HHS. The main reason may be that only one study was included in the subgroup analysis. Salpakoski^26^ point out that there are more patients with difficulty walking after internal fixation than hemiarthroplasty or total hip arthroplasty. Early postoperative walking ability is a significant predictor of postoperative survival in elderly patients with hip fractures^27^. Partial weight‐bearing can be carried out earlier in the hemiarthroplasty group than in the internal fixation group. Kim^12^ points out that most of the patients (140/168) in the hemiarthroplasty group can start to walk with crutches or with a walker within 1 week after the hemiarthroplasty. Early partial weight‐bearing can reduce complications, such as bedsores and decreased lung function, caused by prolonged bedrest^28^.

Implant‐related complications mainly include implant loosening, implant stimulation, cut‐out, prosthesis dislocation, re‐fracture, shortening, protrusion of neck screw, and breakage of the screw. In this meta‐analysis, the implant‐related complications in the internal fixation group were significantly higher than those in the hemiarthroplasty group. Screw cut‐out is the most common complication in the internal fixation group, while prosthesis dislocation is the most common in the hemiarthroplasty group. In Nie's^29^ meta‐analysis, he also points out that hip joint replacement has fewer implant‐related complications than intramedullary fixation in the treatment of intertrochanteric fractures. Most reoperations are due to implant‐related complications. There are many factors affecting reoperation; despite the need for reoperation, some patients still refuse a reoperation. Authen points^30^ out that doctors with less than 3 years of clinical experience have a higher revision rate than clinicians with more than 3 years of clinical experience. In this study, there was no significant difference in the rate of reoperation between the internal fixation group and the hemiarthroplasty group.

Deep venous thrombosis is a problem that cannot be ignored in hip fractures. Mula points out that the probability of DVT after hip fracture is 0.55%. Suspected DVT should be detected by Doppler ultrasonography in time^31^. The perioperative period is crucial for the prevention of DVT; the use of warfarin, low molecular weight heparin, and elastic socks can effectively reduce the incidence of venous embolism. The occurrence of venous embolism will increase the cost of hospitalization^32^. In this meta‐analysis, there was no significant difference in the incidence of DVT between the two groups. In most of the included studies, there is no detailed information on how to prevent venous thrombosis.

With the development of artificial joint technology, hemiarthroplasty is being used increasingly in unstable intertrochanteric fractures in the elderly; most of time the results are satisfactory^33^. The main reason is that artificial joint replacement can obtain anatomical, physiological, and stable joints^34^. Most of the elderly suffer from osteoporosis; internal fixation often can not achieve a stable fixation and can easily to lead to complications. Therefore, hemiarthroplasty has some advantages for unstable intertrochanteric fractures in the elderly: it can reduce postoperative complications, allows early weight‐bearing, and achieves a stable fixation. However, hemiarthroplasty has the disadvantages of potential enormous trauma, longer operation time, and greater intraoperative bleeding volume. The service life of prostheses is limited, which will increase the risk of reoperation.

## Limitations of the Study

Only two RCT were included in the study; seven articles were non‐RCT. Moreover, surgeons have different clinical experience and use different techniques and the follow‐up time varies. The range of ages of the elderly in the studies is large and and they have different physical health conditions, which may lead to high heterogeneity. There are too few studies (<10) to make an adequate evaluation of publication bias. In future clinical studies, it is necessary to conduct a large sample, multicenter study on the therapeutic effects of internal fixation *versus* hemiarthroplasty in the treatment of unstable intertrochanteric fractures in the elderly, to draw more reliable conclusions.

### *Conclusion*

Compared with internal fixation, the hemiarthroplasty group can carry out weight‐bearing training early and has reduced implant‐related complications, but the operation time is longer and intraoperative blood loss is greater. There is no difference in mortality, DVT, reoperation, length of hospital stay, and infection. Hemiarthroplasty may be a better choice for unstable intertrochanteric fractures in the elderly.

## References

[1] Veronese N , Maggi S . Epidemiology and social costs of hip fracture. Injury, 2018, 49: 1458–1460. 29699731 10.1016/j.injury.2018.04.015

[2] Mazzucchelli Esteban R , Perez‐Fernandez E , Crespi‐Villarias N , *et al* Trends in osteoporotic hip fracture epidemiology over a 17‐year period in a spanish population: Alcorcon 1999‐2015. Arch Osteoporos, 2017, 12: 84. 28956291 10.1007/s11657-017-0376-6

[3] Bhattacharya A , Watts NB , Dwivedi A , Shukla R , Mani A , Diab D . Combined measures of dynamic bone quality and postural balance—a fracture risk assessment approach in osteoporosis. J Clin Densitom, 2016, 19: 154–164. 25936482 10.1016/j.jocd.2015.03.005 PMC6894175

[4] Knobe M , Gradl G , Ladenburger A , Tarkin IS , Pape HC . Unstable intertrochanteric femur fractures: is there a consensus on definition and treatment in Germany?. Clin Orthop Relat Res, 2013, 471: 2831–2840. 23389806 10.1007/s11999-013-2834-9 PMC3734428

[5] Camurcu Y , Cobden A , Sofu H , *et al* What are the determinants of mortality after cemented bipolar hemiarthroplasty for unstable intertrochanteric fractures in elderly patients?. J Arthroplasty, 2017, 32: 3038–3043. 28550964 10.1016/j.arth.2017.04.042

[6] Kumar P , Rajnish RK , Sharma S , Dhillon MS . Proximal femoral nailing is superior to hemiarthroplasty in ao/ota a2 and a3 intertrochanteric femur fractures in the elderly: a systematic literature review and meta‐analysis. Int Orthop, 2020, 44: 623–633. 31201487 10.1007/s00264-019-04351-9

[7] Li AB , Zhang WJ , Wang J , Guo WJ , Wang XH , Zhao YM . Intramedullary and extramedullary fixations for the treatment of unstable femoral intertrochanteric fractures: a meta‐analysis of prospective randomized controlled trials. Int Orthop, 2017, 41: 403–413. 27722824 10.1007/s00264-016-3308-y

[8] Moher D , Liberati A , Tetzlaff J , Altman DG . Preferred reporting items for systematic reviews and meta‐analyses: the PRISMA statement. Ann Intern Med, 2009, 151: 264–269. 19622511 10.7326/0003-4819-151-4-200908180-00135

[9] Elhadi AS , Gashi YN . Unstable intertrochanteric fracture in elderly patients: outcome of primary cemented bipolar hemiarthroplasty versus internal fixation. SA Orthop J, 2018, 17: 22–26. 10.5704/MOJ.1803.007 PMC5920257 29725511

[10] Jolly A , Bansal R , More AR , Pagadala MB . Comparison of complications and functional results of unstable intertrochanteric fractures of femur treated with proximal femur nails and cemented hemiarthroplasty. J Clin Orthop Trauma, 2019, 10: 296–301. 30828197 10.1016/j.jcot.2017.09.015 PMC6383068

[11] Kayali C , Agus H , Ozluk S , Sanli C . Treatment for unstable intertrochanteric fractures in elderly patients: internal fixation versus cone hemiarthroplasty. J Orthop Surg, 2006, 14: 240–244. 10.1177/230949900601400302 17200522

[12] Kim JW , Shon HC , Song SH , Lee YK , Koo KH , Ha YC . Reoperation rate, mortality and ambulatory ability after internal fixation versus hemiarthroplasty for unstable intertrochanteric fractures in elderly patients: a study on korean hip fracture registry. Arch Orthop Trauma Surg, 2020. [Epub ahead of print]. 10.1007/s00402-020-03345-2 31970505

[13] Kim SY , Kim YG , Hwang JK . Cementless calcar‐replacement hemiarthroplasty compared with intramedullary fixation of unstable intertrochanteric fractures. A prospective, randomized study. J Bone Joint Surg Am, 2005, 87: 2186–2192. 16203881 10.2106/JBJS.D.02768

[14] Kim YS , Hur JS , Hwang KT , Choi IY , Kim YH . The comparison of compression hip screw and bipolar hemiarthroplasty for the treatment of ao type A2 intertrochanteric fractures. Hip Pelvis, 2014, 26: 99–106. 27536566 10.5371/hp.2014.26.2.99 PMC4971123

[15] Park BJ , Cho HM , Min WB . A comparison of internal fixation and bipolar hemiarthroplasty for the treatment of reverse oblique intertrochanteric femoral fractures in elderly patients. Hip Pelvis, 2015, 27: 152–163. 27536619 10.5371/hp.2015.27.3.152 PMC4972720

[16] Shen J , Wang DL , Chen GX , *et al* Bipolar hemiarthroplasty compared with internal fixation for unstable intertrochanteric fractures in elderly patients. J Orthop Sci, 2012, 17: 722–729. 22868700 10.1007/s00776-012-0272-2

[17] Zhou S , Liu J , Zhen P , *et al* Proximal femoral nail anti‐rotation versus cementless bipolar hemiarthroplasty for unstable femoral intertrochanteric fracture in the elderly: a retrospective study. BMC Musculoskelet Disord, 2019, 20: 500. 31664982 10.1186/s12891-019-2793-8 PMC6820901

[18] Cobden A , Camurcu Y , Duman S , Kocabiyik A , Kıs M , Saklavcı N . Mid‐term survivals of cemented calcar‐replacement bipolar hemiarthroplasty for unstable intertrochanteric fractures in elderly patients. Injury, 2019, 50: 2277–2281. 31630779 10.1016/j.injury.2019.10.023

[19] Fan L , Dang X , Wang K . Comparison between bipolar hemiarthroplasty and Total hip arthroplasty for unstable intertrochanteric fractures in elderly osteoporotic patients. PLoS One, 2012, 7: e39531. 22745778 10.1371/journal.pone.0039531 PMC3382155

[20] Yang S , Liu Y , Yang T , Zou J , Yang H . Early clinical efficacy comparison study of Gamma3 nail, percutaneous compression plate (PCCP) and femoral head replacement (FHR) treatment on senile unstable intertrochanteric fractures. J Invest Surg, 2018, 31: 130–135. 28340311 10.1080/08941939.2017.1282558

[21] Kurmann A , Vorburger SA , Candinas D , Beldi G . Operation time and body mass index are significant risk factors for surgical site infection in laparoscopic sigmoid resection: a multicenter study. Surg Endosc, 2011, 25: 3531–3534. 21638185 10.1007/s00464-011-1753-7

[22] Wang Y , Cui H , Zhang D , Zhang P . Hospitalisation cost analysis on hip fracture in China: a multicentre study among 73 tertiary hospitals. BMJ Open, 2018, 8: e019147. 10.1136/bmjopen-2017-019147 PMC5922469 29703850

[23] Yoo J , Lee JS , Kim S , *et al* Length of hospital stay after hip fracture surgery and 1‐year mortality. Osteoporos Int, 2019, 30: 145–153. 30361752 10.1007/s00198-018-4747-7

[24] Morri M , Ambrosi E , Chiari P , *et al* One‐year mortality after hip fracture surgery and prognostic factors: a prospective cohort study. Sci Rep, 2019, 9: 18718. 31822743 10.1038/s41598-019-55196-6 PMC6904473

[25] Soderman P , Malchau H . Is the Harris hip score system useful to study the outcome of total hip replacement?. Clin Orthop Relat Res, 2001, 384: 189–197. 10.1097/00003086-200103000-00022 11249165

[26] Salpakoski A , Kallinen M , Kiviranta I , *et al* Type of surgery is associated with pain and walking difficulties among older people with previous hip fracture. Geriatr Gerontol Int, 2016, 16: 754–761. 26178923 10.1111/ggi.12552

[27] Iosifidis M , Iliopoulos E , Panagiotou A , Apostolidis K , Traios S , Giantsis G . Walking ability before and after a hip fracture in elderly predict greater long‐term survivorship. J Orthop Sci, 2016, 21: 48–52. 26755386 10.1016/j.jos.2015.09.009

[28] Kuru T , Olcar HA . Effects of early mobilization and weight bearing on postoperative walking ability and pain in geriatric patients operated due to hip fracture: a retrospective analysis. Turk J Med Sci, 2020, 50: 117–125. 31742370 10.3906/sag-1906-57

[29] Nie B , Wu D , Yang Z , *et al* Comparison of intramedullary fixation and arthroplasty for the treatment of intertrochanteric hip fractures in the elderly: a meta‐analysis. Medicine, 2017, 27: e7446. 10.1097/MD.0000000000007446 PMC5502185 28682912

[30] Authen AL , Dybvik E , Furnes O , Gjertsen JE . Surgeon's experience level and risk of reoperation after hip fracture surgery: an observational study on 30,945 patients in the norwegian hip fracture register 2011‐2015. Acta Orthop, 2018, 89: 496–502. 29860911 10.1080/17453674.2018.1481588 PMC6202762

[31] Mula V , Parikh S , Suresh S , Bottle A , Loeffler M , Alam M . Venous thromboembolism rates after hip and knee arthroplasty and hip fractures. BMC Musculoskelet Disord, 2020, 21: 95. 32050949 10.1186/s12891-020-3100-4 PMC7017506

[32] Trivedi NN , Sivasundaram L , Wang C , *et al* Chemoprophylaxis for the hip fracture patient: a comparison of warfarin and low‐molecular‐weight heparin. J Orthop Trauma, 2019, 33: 216–219. 31008818 10.1097/BOT.0000000000001435

[33] Cui Q , Liu YS , Li DF , *et al* Cemented hip hemiarthroplasty clinical observations on unstable intertrochanteric fracture in elderlies. Eur J Trauma Emerg Surg, 2016, 42: 651–656. 26351080 10.1007/s00068-015-0566-0

[34] Xie Y , Zhou H . Primary cemented hemiarthroplasty for unstable intertrochanteric fractures in elderly severe osteoporotic patients. Injury, 2020, 16: 34. 10.1016/j.injury.2020.01.010 31928710

